# Lipidomics: An Evolving Discipline in Molecular Sciences

**DOI:** 10.3390/ijms16047748

**Published:** 2015-04-08

**Authors:** Giuseppe Astarita, Mario Ollero

**Affiliations:** 1 Waters Corporation, Milford, MA 01757, USA; 2 Department of Biochemistry and Molecular & Cellular Biology, Georgetown University, Washington, DC 20515, USA; 3 Université Paris Est—Créteil Val-de-Marne, Créteil 94010, France; 4 INSERM, U955, Créteil 94010, France

Recent advances in technologies for lipid analysis have contributed to the consolidation of lipidomics as a distinct discipline in molecular sciences [1,2,3,4,5,6]. This technological development has paralleled the discovery of a broad range of essential cellular functions associated with lipids, from cell-signaling and membrane dynamics to intercellular communication and the regulation of gene expression and immune response [7].

Lipids are present in living organisms that span the spectrum of biological complexity: animals, plants, fungi, protists, bacteria, archaea, and viruses [1,2,6]. In humans, alterations in lipid metabolites are associated with various human diseases including obesity, heart disease, and diabetes [4]. Therefore, global lipid analysis—lipidomics—has become a powerful research tool. As such, in the area of personalized medicine, lipidomics is used to investigate biological mechanisms underlying diseases. Lipidomics is also playing a significant role in the discovery of new therapeutic targets and biomarkers of health and disease. Additional areas of interest for lipidomics are plant, microbial, and nutritional research [8].

Comprehensive analyses of the wide array of lipids in biological samples proves challenging primarily for analytical chemistry. These complex mixtures of lipids include many, diverse chemical structures and a large, dynamic range of concentrations. Consequently, interest in adapting novel technologies for lipid analysis continues undiminished. The study of lipid biology is, therefore, undergoing a remarkable, technology-driven transformation that involves, most notably, mass spectrometry (MS) and its ancillary techniques such as liquid chromatography and ionization sources [9,10].

As we progress to a new era of lipid analysis, the potential to accurately and rapidly measure hundreds of individual molecular species provides the opportunity to use more complex lipid profiles for drug discovery and for disease diagnostics and prognosis.

## 1. The Evolution of Lipid Analysis

Our knowledge of lipid biology has been hampered by analytical limitations. The development, in the late 1980s, of electrospray ionization (ESI) for MS revolutionized the study of lipids. ESI enabled the introduction into a mass spectrometer of nonvolatile lipid species, from the liquid phase directly into the gas phase, without the need for prior derivatization. ESI-MS, therefore, replaced some of the immunological, radioactive, fluorometric, and colorimetric techniques used in traditional laboratory analysis. The parallel development of mass-spectrometric techniques proved itself a sensitive tool for identifying, characterizing, and quantifying lipid species.

## 2. Lipidomic Approaches

Currently, three main approaches characterize lipidomics research:

(1) *Untargeted Lipidomics* offers the capability to explore, in an unbiased fashion, the lipid composition of a sample [9,11]. Comparative, untargeted analysis offers investigators the opportunity to identify new biomarkers or previously unknown mechanistic pathways involved in health and disease or in nutrition. This exploration can yield, for example, unexpected discoveries of particular lipids involved in certain pathologies.
(2) *Targeted Lipidomics* aims to monitor selected lipids [12]. It can be used to validate initial discoveries or for routine analysis in clinical research. Compared to untargeted approaches, targeted approaches can enhance analytical sensitivity. Such enhancement is often required before analyzing lipids like eicosanoids, which are available only in very low abundance.
(3) *MS Lipidomics Imaging* and *in situ* lipidomics provide spatial information about the lipid composition in tissues—a sort of molecular microscope [13,14]. At the core of this approach is the use of a desorption ionization tool, including matrix assisted laser desorption ionization (MALDI), desorption electrospray ionization (DESI), and secondary ions mass spectrometry (SIMS). The use of other ambient ionization tools, including rapid evaporative ionization mass spectrometry (REIMS) and direct analysis in real time (DART) allow rapid, real-time screenings of lipids for predictive, preventive, and personalized medicine.

## 3. Challenges Ahead

The identification of isobaric and isomeric species, which are not resolvable solely by accurate mass, remains a challenge for MS-based lipidomics. Orthogonal technologies, such as chromatography and ion mobility, can be integrated to assist MS detection. The result of this integration is more confident lipid identification in the assignment of the chemical structure. For example, monodimensional or multidimensional liquid chromatography (LC) can improve lipidomic separations before MS detection [15,16,17,18]. In addition, supercritical fluid chromatography (SFC), which uses liquid CO_2_ as mobile phase, enables alternative ways of separating lipids [19,20,21,22,23,24]. Innovative, microfluidic applications offer the advantages of increased sensitivity and lower injection volume over traditional LC. Both SFC and microfluidic technologies operate at an environmentally friendly, cost-saving scale with respect to solvent consumption and waste disposal.

In addition to chromatography, the use of ion mobility can support traditional LC-MS lipidomic protocols by enabling the measurement of collision-cross sections, a measure of the shape of molecules that can serve as an additional coordinate for identifying lipids [25]. Novel informatics solutions permit us to process and mine ion-mobility-enriched information, in a user-friendly way, to deliver meaningful results from complex lipidomic analyses. A post-ionization separation tool like ion mobility becomes particularly useful when no chromatographic separation occurs before MS detection, the case with the MS techniques of desorption-ionization and ambient-ionization.

## 4. New Technology Is Bringing Innovation in Lipidomics

The application areas of lipidomics have been continually growing, as more researchers seek to harness the combined power of technology and our understanding of lipid biology. Lipidomics is already revolutionizing lipid research. We now can amass certain types of experimental data about lipid composition that is 100 or, in some cases, even 1000 times faster than we could 20 years ago. The resultant volume of information offers an unprecedented opportunity to describe biological systems and thus lend itself to unexpected discoveries. Lipidomic research is already helping to elucidate disease mechanisms and biological markers for health and disease.

## 5. Future Directions

We expect that software solutions will allow us to integrate lipidomics data with multi-disciplinary information, including genomic and proteomic data, for pathway analysis. The standardization of procedures for sample preparation and analysis will increase inter-laboratory reproducibility, allowing us to share and compare data across the globe. The possibility for developing standard procedures for analyzing lipids could lead to the development of lipidomic kits, facilitating routine analysis for quality control and clinical research.

In this special issue, we present a snapshot of the wide array of studies being undertaken using lipidomic approaches. This collection of papers can offer a glimpse of the potential of lipidomics approaches in biomedical research and in food and nutrition research.

Finally, we would like to express our deep gratitude to each of the authors for their contribution and support in producing this special issue addressing bioactive lipids and lipidomics.
